# The effect of alexithymia on self-perceived aging among community-dwelling older adults with multiple chronic conditions: the mediating role of maladaptive cognitive emotion regulation strategies

**DOI:** 10.3389/fpsyt.2024.1437478

**Published:** 2024-11-08

**Authors:** Jianou Xu, Bin Shang, Jun Zhang, Caifeng Luo, Zekun Bian, Fei Lv, Zhengxia Yang

**Affiliations:** ^1^ School of Medicine, Jiangsu University, Zhenjiang, Jiangsu, China; ^2^ Operating Room, The First People’s Hospital of Lianyungang, Lianyungang, Jiangsu, China; ^3^ Affiliated Hospital of Jiangsu University, Zhenjiang, Jiangsu, China; ^4^ Department of Nursing, Jiangsu University Jingjiang College, Zhenjiang, Jiangsu, China; ^5^ The First Affiliated Hospital of Wannan Medical College, Wuhu, Anhui, China

**Keywords:** alexithymia, maladaptive cognitive emotion regulation, multiple chronic conditions, self-perceived aging, older adults

## Abstract

**Objectives:**

This study investigated the mediating role of maladaptive cognitive emotion regulation strategies in the relationship between alexithymia and self-perceived aging among older adults.

**Methods:**

We surveyed 478 Chinese community-dwelling older adults from November 2022 to May 2023. The Toronto Alexithymia Scale (TAS-20), Cognitive Emotion Regulation Questionnaire (CERQ), and Brief Aging Perceptions Questionnaire (B-APQ) were used. Correlation analyses, multiple linear regression analysis, and structural equation modeling were performed.

**Results:**

The average age of participants was 71.52 ± 7.80 years, and the number of chronic diseases was distributed as follows: 270 (56.49%) had 2 disease conditions, 156 (32.64%) had 3 disease conditions, and 52 (10.88%) had 4 disease conditions or more. Scores for alexithymia 57.83 ± 10.19; maladaptive cognitive emotion regulation strategies 49.63 ± 10.65; and self-perceived aging 58.74 ± 10.23. Alexithymia and maladaptive cognitive emotion regulation strategies were positively correlated with negative self-perceived aging (r=0.665 and r=0.673, respectively), explaining 51.8% of the variance in self-perceived aging. Structural equation modeling results showed that alexithymia had a direct effect (of 0.368) on self-perceived aging, accounting for 44.1% of the total effect. Maladaptive cognitive emotion regulation strategies partially mediated the relationship, with a mediation effect of 0.386, accounting for 51.8% of the total effect.

**Conclusion:**

These findings suggest that addressing maladaptive cognitive emotion regulation strategies can help reduce negative self-perceived aging in elderly individuals with multiple chronic conditions, particularly among those with alexithymia.

## 1 Introduction

With increased population aging, multiple chronic conditions (MCC) have emerged as an important global public health concern. The biopsychological challenges that accompany MCC have a profound impact on the journey toward healthy aging (1). Self-perceived aging (SPA) is a crucial indicator for evaluating the process of healthy aging, encompassing the subjective perception and emotional responses of older individuals to the physiological, psychological, and social challenges of aging (2). According to stereotype embodiment theory (3), individuals develop age-related stereotypes long before reaching old age, and these beliefs become internalized as they aging. When society categorizes individuals as elderly, these age stereotypes influence their views of aging (VoA), significantly influencing their biopsychosocial well-being. Positive SPA plays a pivotal role in promoting healthy aging among older individuals (4, 5). Negative SPA increases risk of stroke and cardiovascular disease (6). Studies have indicated that older adults with MCC are more likely to experience disrupted emotions than those with a single chronic condition (7, 8). Older adults with MCC who have negative emotions often experience physical illness or certain psychological experiences such as a negative SPA (9), which is exactly what these older adults are concerned about. Older adults with MCC who have negative SPA are more likely to be frail (10). However, there is limited research on the SPA of older adults with MCC. Understanding the mechanisms that impact SPA in this populations and implementing targeted interventions to mitigate their negative SPA may be important for reducing the risk of frailty and disease, promoting accurate self-awareness, and enhancing quality of life in late adulthood (1).

SPA, as a form of self-awareness, is susceptible to the influence of several factors (11–13). Alexithymia refers to difficulty in recognizing and describing emotions, as well as differentiating them from physical sensations (14). Studies have shown that alexithymia is significantly present in patients with chronic diseases such as asthma (15), diabetes (16), and cancer (17), and that alexithymia negatively affects patients’ accurate perceptions of social support and disease management. Okanli et al. (18) found that alexithymia affects disease perception in cancer patients. Karpuz Seren et al. (19) discovered that difficulty in identifying and expressing emotions due to alexithymia negatively impacts self-perceptions and perceptions of others in multiple sclerosis patients, leading to social limitations. These studies suggest that alexithymia may exaggerate the negative self-perception of somatic aspects (20), implying that alexithymia might have a unfavorable impact on SPA in older individuals with MCC. Research has shown that neurocognitive abilities, which are closely related to age, are highly negatively correlated with alexithymia (21). Alexithymia has a high prevalence among older adults. The prevalence of alexithymia among older persons in Finland has been reported to be 29.3% (22). In China, the prevalence of alexithymia in community-dwelling older adults with chronic diseases ranges from 44.34-49.4% (23). Tian et al. (24) reported that aging attitudes of older adults in nursing homes were negatively associated with alexithymia. However, the relationship between alexithymia and SPA in older adults with MCC needs to be explored. The extent to which alexithymia predicts SPA and the predictive pathways warrant further investigation.

Cognitive emotion regulation strategies refer to the ways that individuals cope with stress and emotional distress in an environmental context, and are broadly categorized into adaptive and maladaptive regulation strategies (25). Maladaptive regulation strategies include self-blame, rumination, catastrophizing, and blaming others. Previous studies have shown that individuals with high alexithymia are more likely to use maladaptive regulation strategies (20, 26). Shang et al. (27) based on network analysis found that catastrophizing is the core node and rumination is the bridge node in the network of alexithymia and cognitive emotions in older adults with MCC. Catastrophizing refers to the idea that individuals over-enforce their fearfulness, and catastrophizing may amplify concerns about aging and lead to a more negative VoA. Lv et al. (28) found that community-dwelling older adults with alexithymia had difficulty effectively processing and regulating their emotions. When encountering emotional disorders, they were more inclined to use maladaptive cognitive emotion regulation strategies such as rumination and self-blame. These maladaptive emotion regulation strategies exacerbated individuals’ negative cognitions and self-perception (29), which may have negative impact on the SPA in the elderly. Although these links have been partially established, it remains to be verified whether maladaptive cognitive emotion regulation strategies play a key mediating effect in alexithymia and SPA in older adults.

Therefore, this study aimed to investigate the relationships among alexithymia, cognitive emotion regulation strategies, and SPA in community-dwelling older adults with MCC and to understand the links between emotion regulation strategies with SPA, especially in the alexithyimia context, and its likely healthy impact on aging and multimorbidity. Our specific hypotheses are as follows. 1: Alexithymia will affect the SPA of community-dwelling older adults with MCC. 2: Maladaptive cognitive emotion regulation strategies will mediate the relationship between alexithymia and negative SPA in older adults with MCC. To our knowledge, this is the first study to evaluate the mediating role of maladaptive cognitive emotion regulation strategies in the relationship between alexithymia and negative SPA among community-dwelling older Chinese adults with MCC.

## 2 Methods

### 2.1 Study design and participants

This study used a cross-sectional, descriptive and analytical survey. From November 2022 to May 2023, a convenience sampling method was used to recruit elderly individuals with MCC from communities and villages in four prefecture-level cities in Jiangsu Province, China (Nanjing, Zhenjiang, Lianyungang, and Suqian). Participants were recruited at community health centers and community activity centers. The types of chronic diseases were determined using the Charlson Comorbidity Index (30) combined with geriatrics expert advice, resulting in the inclusion of 20 common chronic diseases: coronary artery disease, congestive heart failure, chronic pulmonary disease, peptic ulcer disease, peripheral vascular disease, mild liver disease, cerebrovascular disease, connective tissue diseases, diabetes, hypertension, dementia, hemiplegia, diabetes with organ damage, moderate to severe renal disease, tumors within 5 years, leukemia, lymphoma, moderate to severe liver disease, metastatic solid tumors and AIDS. Elderly patients with two or more diseases are considered to be elderly patients with MCC. The inclusion criteria were as follows: (1) aged ≥ 60 years; (2) two or more chronic diseases; and (3) provision of informed consent and voluntary participation in the study. The exclusion criteria were as follows: (1) cognitive impairment or intellectual issues that may affect the validity of informed consent for this study, (2) not community-dwelling (permanent residence < 6 months) or with an unknown place of residence, or (3) participation in other research projects. It should be noted that determining that the participants had cognitive impairment and intellectual problems was not done through relevant scales such as the Mini-Mental State Examination (MMSE), but by judging that they incorrectly answered the most basic numerical or common sense responses. This study was approved by the Medical Ethics Committee of Jiangsu University (No. 20221019-7) and conformed to the provisions the Declaration of Helsinki. All respondents completed the questionnaire with informed consent.

We employed a combination of online and offline methods to ensure comprehensive data collection, a strategy supported by previous research to minimize the risk of missing crucial values (31). Questionnaires were administered by uniformly trained researchers to older adults with MCC who met the inclusion criteria, and this survey was voluntary and anonymous. Online data collection took place on the Wenjuanxing platform (www.wjx.cn), where older participants filled out the questionnaire online using their cell phones. where elderly participants independently completed questionnaires. If elderly individuals were illiterate or faced challenges in completing the online survey, researchers provided verbal assistance and then transcribed their responses accurately into the online platform after verification. To accommodate elderly participants without smartphones who preferred to complete the questionnaire independently, paper-based questionnaires mirroring the online version were also made available, with consistent data entry requirements. Prior to the questionnaire, the purpose of the survey and the principle of voluntariness were introduced to all participants, and respondents completed the questionnaire to indicate their informed consent. Following questionnaire completion, two researchers conducted a meticulous cross-check of the responses. The online survey datas were exported by the Wenjuanxing platform (https://www.wjx.cn/) after two-person verification, and the face-to-face survey datas were also verified by two people. In order to ensure the data accuracy, questionnaires with obvious same answers or wavy answers, indicating that respondents may not have answered carefully, were considered invalid. Out of the 515 questionnaires initially collected, 37 (7.18%) were found to be invalid and were subsequently removed. This process resulted in the retention of 478 valid questionnaires, achieving an impressive effective response rate of 92.82%.

### 2.2 Measurements

#### 2.2.1 Demographic information and diseases characteristics

The questionnaire was developed by the research team and included items related to gender, age, marital status, level of education, average monthly household income, place of residence, residential status, number of chronic diseases, types of medications, health care payment methods, economic burden of diseases, occurrence of acute events in the past year, and self-perceived health status.

#### 2.2.2 The Toronto Alexithymia Scale

The scale was originally developed by Taylor (14) and was later adapted to create a Chinese version by Yi et al. (32). The Chinese version of the scale had a Cronbach’s α coefficient of 0.830. It consists of 20 items divided into three dimensions: difficulty identifying feelings, difficulty describing feelings, and externally oriented thoughts. Items are rated on a 5-point Likert scale, ranging from 1 (strongly disagree) to 5 (strongly agree). Items 4, 5, 10, 18, and 19 are reverse-scored. The total score ranges from 20 to 100, with higher scores indicating a higher level of alexithymia. In this study, the total scale had a Cronbach’s α coefficient of 0.890, and the Kaiser-Meyer-Olkin value was 0.935.

#### 2.2.3 The Brief Aging Perceptions Questionnaire

The scale was originally developed by Sexton et al. (2) and was later adapted to create a Chinese version by Hu et al. (33); the Chinese version was used in a survey of community-dwelling elderly individuals and had a Cronbach’s α coefficient of 0.758. The scale comprises 17 items divided into five dimensions: negative outcome and control, positive outcome, chronic timing, positive control, and emotional representation. Items are rated on a 5-point Likert scale, ranging from 1 (strongly disagree) to 5 (strongly agree). Items 4-6 and 8-10 are reverse-scored. The total score is calculated by summing all items and ranges from 17 to 85, with higher scores indicating more negative SPA. In this study, the total scale had a Cronbach’s α coefficient of 0.883, and the Kaiser-Meyer-Olkin value was 0.888.

#### 2.2.4 The Cognitive Emotion Regulation Questionnaire

The scale was originally developed by Garnefski et al. (25) and was later adapted to create a Chinese version by Zhu et al. (34). The Chinese version of the scale had a Cronbach’s α coefficient of 0.667. It is divided into two subscales: adaptive regulation strategies and maladaptive regulation strategies. In this study, only the Maladaptive Cognitive Emotion Regulation Strategies subscale (CERQ_M) was used, which includes four dimensions: self-blame, rumination, catastrophizing, and blaming others. It has a total of 16 items. Items are rated on a 5-point Likert scale, with values ranging from 1 (‘never’) to 5 (‘always’). The total score ranges from 16 to 80, with higher scores indicating a greater tendency to use maladaptive regulation strategies when facing negative events. In this study, the scale had a Cronbach’s α coefficient of 0.931, and the Kaiser-Meyer-Olkin value was 0.899.

### 2.3 Statistical analysis

Descriptive statistics and correlation analysis were conducted using SPSS 26.0. The quantitative data were normally distributed and reported using mean and standard deviation. Pearson correlation analysis was performed to examine the relationships of relevant variables. AMOS 24.0 was employed to construct a structural equation model and test for mediating effects. The model fit was assessed by the following indices: chi-square/degrees of freedom (χ²/df) between 1 and 3 signifies the hypothesized model fits the sample data well; comparative fit index (CFI), incremental fit index (IFI), and goodness-of-fit index (GFI) values above 0.90 indicate good fit; and root mean square error of approximation (RMSEA) less than 0.08 suggests a close fit (35). The bootstrap method was used to examine the mediation model, with a significance level of α = 0.05.

## 3 Results

### 3.1 General characteristics of the participants

A total of 478 valid questionnaires were analyzed. The average age of respondents was 71.52 ± 7.80 years. General characteristics are shown in **Table 1**.

**Table 1:** General characteristics (n = 478).

Variable	Group	Total *N* (%)
Gender	Male	263 (55.02%)
	Female	215 (44.98%)
Marital status	Married Single(unmarried/ widowed)	404 (84.52%) 74 (15.48%)
Education levels	Primary school or below	202 (42.26%)
	Junior high school Senior high school or above	179 (37.45%) 97 (20.30%)
Monthly income (RMB)	≤3000	202 (42.26%)
	3000-5000	165 (34.52%)
	5000-8000	91 (19.04%)
	≥8000	20 (4.18%)
Place of residence	Rural	306 (64.02%)
	Urban	172 (35.98%)
Number of chronic diseases	2	270 (56.49%)
	3	156 (32.64%)
	≥4	52 (10.88%)
Types of medication	No	20 (4.18%)
	1-2	230 (48.12%)
	3-4	167 (34.94%)
	≥5	61 (12.76%)
Health care payment	Out-of-pocket or had Commercial insurance	21 (4.39%)
	Rural insurance	299 (62.55%)
	Employee medical Insurance	67 (14.02%)
	Urban resident medical Insurance	91 (19.04%)
Economic burden of diseases	Mild	87 (18.20%)
	Moderate	321 (67.15%)
	Severe	70 (14.64%)
Acute events in the past year (including hospitalization, surgery, falls, etc.)	No	288 (60.25%)
	Yes	190 (39.75%)
Self-perceived health status	Poor	132 (27.62%)
	Fair	256 (53.56%)
	Good	90 (18.83%)

### 3.2 Correlation analyses of alexithymia, maladaptive cognitive emotion regulation strategies, and SPA in elderly individuals with MCC

Among elderly individuals with MCC, the total score for alexithymia was 57.83 ± 10.19, the total score for maladaptive cognitive emotion regulation strategies was 49.63 ± 10.65, and the total score for SPA was 58.74 ± 10.23. Pearson correlation analysis results revealed that negative SPA was positively correlated with alexithymia (r=0.665, P<0.001) and maladaptive cognitive emotion regulation strategies (r=0.673, P<0.001). It was shown that older adults with MCC with higher levels of alexithymia had higher SPA questionnaire scores and tended to have more negative SPA. Similarly, older adults with MCC who tended to use maladaptive cognitive emotion regulation strategies when faced with negative events had a more negative SPA. Alexithymia was positively correlated with maladaptive cognitive emotion regulation strategies (r=0.722, P<0.001). This suggested that older adults with MCC who had higher levels of alexithymia were more likely to use maladaptive cognitive emotion regulation strategies. Specific correlations for each dimension are shown in **Table 2**.

**Table 2:** Correlation analysis of alexithymia, maladaptive cognitive emotion regulation strategies, and SPA in elderly individuals with MCC.

	1	1.1	1.2	1.3	2	2.1	2.2	2.3	2.4	3	3.1	3.2	3.3	3.4	3.5
1. Alexithymia	1														
1.1 Difficulty identifying feelings	.939**	1													
1.2 Difficulty describing feelings	.913**	.839**	1												
1.3 Externally oriented thoughts	.764**	.548**	.555**	1											
2 Maladaptive cognitive emotion regulation strategies	.722**	.717**	.697**	.450**	1										
2.1 Self-blame	.676**	.678**	.639**	.424**	.793**	1									
2.2 Rumination	.359**	.404**	.381**	.112*	.697**	.461**	1								
2.3 Catastrophizing	.618**	.593**	.604**	.410**	.870**	.568**	.457**	1							
2.4 Blaming others	.666**	.635**	.621**	.482**	.860**	.572**	.379**	.756**	1						
3 Self-perceived aging	.665**	.665**	.639**	.412**	.673**	.563**	.458**	.562**	.584**	1					
3.1 Consequences and control-negative	.658**	.658**	.614**	.425**	.614**	.537**	.387**	.506**	.548**	.821**	1				
3.2 Consequences-positive	.106*	.099*	.107*	0.072	.186**	0.088	.197**	.167**	.147**	.500**	.100*	1			
3.3 Timeline-chronic	.530**	.551**	.531**	.270**	.528**	.474**	.351**	.424**	.457**	.707**	.604**	0.025	1		
3.4 Control-positive	.107*	.102*	.124**	0.052	.175**	0.088	.210**	.152**	.118**	.487**	0.048	.679**	0.056	1	
3.5 Emotional representations	.715**	.704**	.666**	.483**	.672**	.613**	.364**	.574**	.609**	.800**	.764**	.104*	.591**	.094*	1

*P<0.05; **P<0.01.

### 3.3 Regression analysis of factors influencing SPA in elderly people with MCC

A multiple linear regression analysis was conducted with SPA in elderly individuals with MCC as the dependent variable and the total scores of alexithymia and maladaptive cognitive emotion regulation strategies as independent variables. The results showed that alexithymia (β=0.376, P<0.001) and maladaptive cognitive emotion regulation strategies (β=0.386, P<0.001) collectively explained 51.8% of the total variation in SPA in elderly individuals with MCC. Both alexithymia scores and maladaptive cognitive emotion regulation strategy scores positively predicted SPA scores, as shown in **Table 3**.

**Table 3:** Multiple linear regression analysis results of factors influencing SPA in elderly people with MCC.

Variable	B (coefficient)	SE (standard error)	β (standardized coefficient)	t	P value
Constant	17.815	1.893	—	9.412	<0.001
Alexithymia	0.376	0.046	0.375	8.160	<0.001
Maladaptive cognitive emotion regulation strategies	0.386	0.044	0.402	8.764	<0.001

R²=0.520, adjusted R²=0.518, F=257.174, P<0.001, Durbin-Watson value=1.615.

### 3.4 The mediating effect of maladaptive cognitive emotion regulation strategies

Harman’s single-factor test was used to check for common method bias, and the results showed that there were 12 factors with eigenvalues greater than 1. The cumulative variance explained by the first factor was 27.2%, which is below the critical threshold (40%). This indicates that the study results were not substantially impacted by common method bias (36).

**Figure 1:**
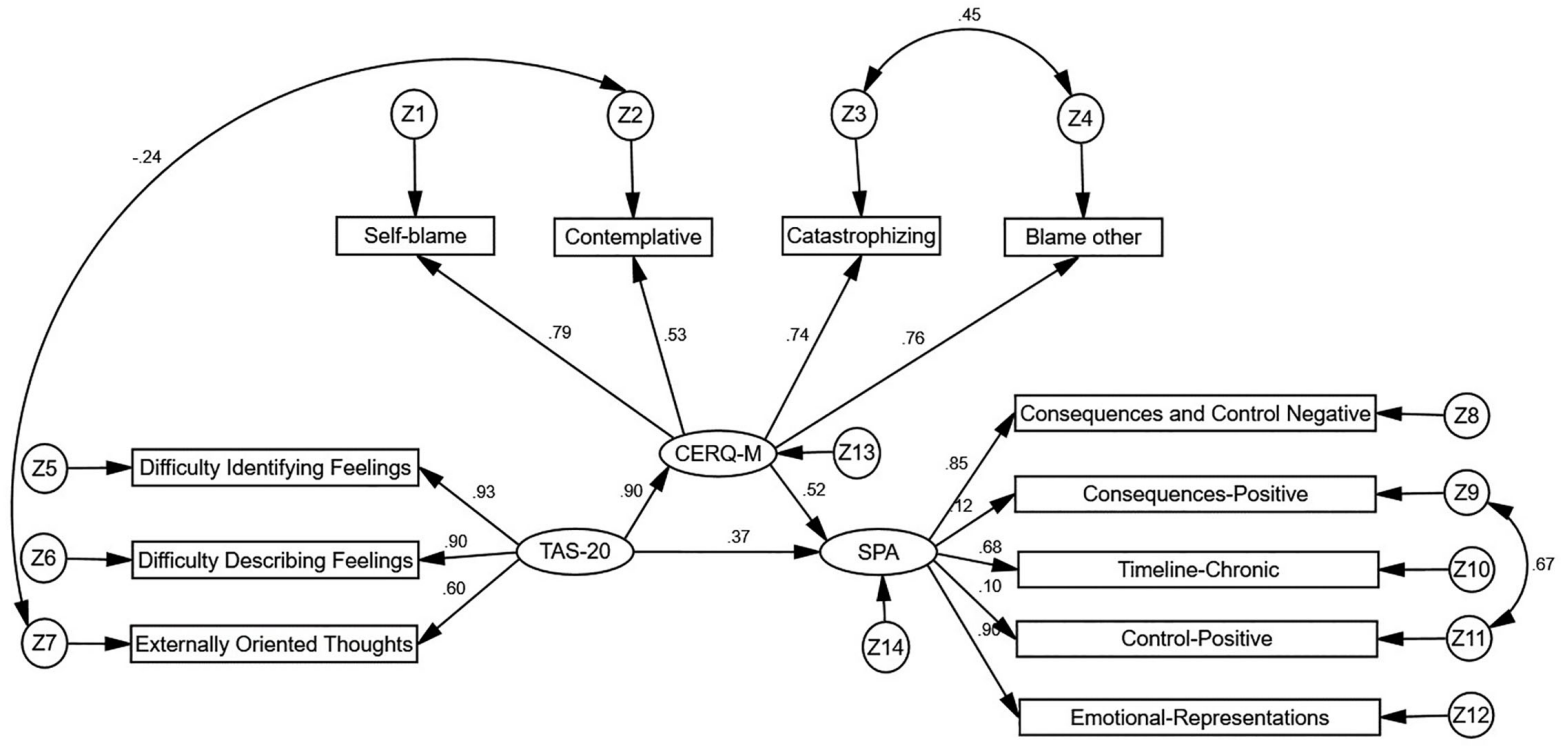
Mediating effect of maladaptive cognitive emotion regulation strategies in the relationship between alexithymia and SPA. TAS-20, alexithymia; CERQ-M, maladaptive cognitive emotion regulation strategies; SPA, self-perceived aging.

Using AMOS 24.0 software, a structural equation model was constructed with SPA as the dependent variable, alexithymia as the independent variable, and maladaptive cognitive emotion regulation strategies as the mediating variable. Maximum likelihood estimation was used to fit the model parameters, and the model was adjusted based on modification indices. All fit indices for the mediation model were within acceptable ranges, indicating a good model fit, as shown in **Figure 1**. The criteria and results for the model fit indices are presented in **Table 4**.

**Table 4:** Fit indices of the mediation model.

Fit indicator	χ2/df	GFI	AGFI	CFI	IFI	TLI	RMSEA
Standard	1-3	>0.9	>0.9	>0.9	>0.9	>0.9	<0.08
Result	2.183	0.965	0.943	0.983	0.984	0.977	0.050

The structural equation modeling results revealed that alexithymia had a direct positive predictive effect on maladaptive cognitive emotion regulation strategies (β=0.896, P<0.001). Maladaptive cognitive emotion regulation strategies had a direct positive predictive effect on negative SPA (β=0.522, P<0.001). Alexithymia had a direct positive predictive effect on negative SPA (β=0.368, P=0.002), accounting for 44.1% of the total effect. Bootstrap analysis was employed to evaluate the mediating effect in the model, with 5000 bootstrap samples and a 95% confidence interval. The results indicated that the 95% confidence interval for the mediating effect of maladaptive cognitive emotion regulation strategies in the relationship between alexithymia and negative SPA was 0.228-0.823; this range excluded 0, indicating a statistically significant partial mediating effect of maladaptive cognitive emotion regulation strategies in the relationship between alexithymia and SPA. The mediating effect value was 0.896×0.522 = 0.467, accounting for 55.9% of the total effect, as shown in **Table 5**.

**Table 5:** Specific results of the mediation analysis.

Project	Effect size	SE	Bootstrapped 95%CI	
			Lower bound	Upper bound
Total effect	0.835	0.023	0.788	0.878
Direct effect	0.368	0.160	0.005	0.622
Indirect effect	0.467	0.155	0.228	0.823

## 4 Discussion

This study explored the potential mechanisms by which alexithymia affect SPA in Chinese community-dwelling older adults with MCC from the perspective of emotion regulation strategies. The findings showed that alexithymia directly affect SPA, and maladaptive cognitive emotion regulation strategies play a significant mediating role in this process. These results not only reveal the importance of psychological factors in SPA, but also suggest that by modulating cognitive emotion regulation strategies, it may have a positive impact on improving the physical and mental health of older adults with MCC, and enhancing their quality of life.

The research results indicate that the SPA score of elderly individuals with MCC (58.74 ± 10.23) is higher (i.e., with more negative implications) than that previously reported by Zhang et al. (37). This suggests that the SPA score in this survey is relatively high. One possible explanation is that all the participants in this survey were elderly individuals with MCC. Among them, nearly half took more than two types of medication, and 70 (14.64%) experienced a heavy economic burden due to their illnesses. The increased disease burden and the use of multiple medications may contribute to their negative SPA (38, 39). The results also showed that 74 (15.48%) elderly individuals lived alone. Older adults living alone who lacked interactive healthy family networks (40) were more prone to frailty as well as negative emotions such as loneliness, depression, and anxiety, which exacerbate the self-perception of aging in such patients (12). Patients with alexithymia, while not usually experiencing unpleasant emotions, tend to manifest them through different somatic symptoms as well as negative cognitions, including negative SPA. Additionally, there may be bidirectional communication of self-perceived health and SPA. Negative SPA links the aging process with more unfavorable consequences, such as relevant physical, psychological, and social losses (41). It was found that the worst self-assessed health status of older adults was negatively associated with their SPA scores (42). Elderly individuals with MCC are more likely to attribute bodily discomfort and functional decline to the aging process (4, 43). In the present study, 132 (27.62%) elderly individuals perceived their health as poor, further confirming these findings. Studies have shown that older adults with negative SPA have shorter survival than their age-matched peers who hold the opposite outlook (44). Therefore, community health care professionals should pay attention to the potential inaccurate SPA of elderly individuals with MCC. Individualized health care plans, education, psychological support, and active aging activities can healthily cope with the aging process (45).

The Pearson correlation analysis results demonstrated a close relationship between alexithymia and negative SPA. The results of the multiple linear regression analysis further confirmed that alexithymia significantly influences SPA. Prior research has also supported this notion, indicating that alexithymia tends to lead to distorted body perceptions and consequently biased cognitions (18) in individuals. Patients with alexithymia may have biased perceptions of themselves and their emotions, possibly overfocusing on their own flaws or issues and incorrectly attributing these problems to aging, thereby reinforcing their negative SPA (20, 46). The negative SPA coupled with alexithymia may make older adults more likely to somatically express emotional conflicts and lead to develop more illnesses. Additionally, it may affect the health of older adults through several biological pathways, including accelerated aging processes mediated through high C-reactive protein (44). Moreover, individuals with alexithymia may also experience social difficulties, potentially impacting their interpersonal relationships, and likely their self-perceptions. (20). Rosenberg et al. (47) revealed that alexithymia is associated with reduced responsiveness of the brain’s cortical regions and medial frontal lobe to satisfaction. This may impair the automatic recognition, integration, and judgment of emotions, resulting in damage to interpersonal relationships linked to alexithymia. It becomes challenging for elderly individuals to establish intimate connections with others, leading to a lack of essential social support and potentially generating feelings of helplessness and failure when confronting the aging process (8). In this study, the total score for alexithymia in elderly individuals with MCC was 57.83 ± 10.19, which is similar to that in previous research in Chinese elderly diabetes hospitalized patients (48), and lower than that of Turkish diabetes hospitalized patients, whose total score of alexithymia was 62.97 ± 10.19 (16). The reasons for this may be related to the fact that in addition to cultural differences, more than half of the hospitalized patients in the Turkish study had other chronic diseases and diabetes-related complications. Overall, these patients exhibit moderate to high levels of alexithymia. Therefore, health care professionals should pay attention to the influence of alexithymia on the SPA of elderly individuals with MCC. Effective intervention measures, such as dialectical behavior therapy (49) and rational emotive therapy (50), should be implemented to improve alexithymia levels, thereby preventing or mitigating the adverse effects of this condition on SPA.

The results of the mediation analysis indicated that maladaptive cognitive emotion regulation strategies play a significant mediating role in the relationship between alexithymia and SPA among elderly individuals with MCC. This implies that alexithymia not only directly influences the SPA of elderly individuals with MCC but also indirectly affects SPA by influencing maladaptive cognitive emotion regulation strategies, with the mediating effect accounting for 55.9% of the total effect. This is the first confirmation of such a mediating effect in elderly Chinese individuals with MCC. Maladaptive cognitive emotion regulation strategies, as a negative subset of cognitive emotion regulation strategies, may render elderly individuals with MCC more vulnerable to adversity, which may consequently affect their VoA (20, 51). A meta-analysis by Rogier et al. (52) showed that maladaptive cognitive emotion regulation strategies are strongly associated with suicide, which is particularly prominent among older adults. This suggests that maladaptive strategies not only affect SPA, but may also increase the risk of more serious psychosocial risks. Multiple studies have confirmed that alexithymia influences an individual’s choice of cognitive emotion regulation strategies (20, 53). The analysis suggests that individuals with alexithymia find it difficult to recognize and express emotions, leading them to overfocus on external matters and making them more susceptible to more negative cognitions and worst coping strategies. This handicaps can trigger maladaptive cognitive emotion regulation strategies such as catastrophizing and self-blame, which may further lead to negative self-perceptions and pessimism (20, 54). Moreover, pessimism is traditional among elderly Chinese individuals, which also increases their susceptibility to pessimistic thoughts. This may lead them to attribute their physical burdens and changes in interpersonal relationships to the aging process (4, 43). Butler et al. (55) noted that cultural context shapes the meaning that individuals give to emotional expressions that occur during social interactions. Chinese culture emphasizes control and tolerance of emotions, and this cultural characteristic may make older adults more inclined to repress emotions. The results of this study may provide a reference for countries with similar cultural characteristics to China. Shang et al. (27) conducted a network analysis and reported that catastrophizing was a core node in the cognitive emotion regulation network of elderly individuals with MCC, while rumination served as a bridging node. This indicates that alexithymia may affect the emotion regulation of elderly individuals by influencing rumination, which in turn impacts their emotion regulation. Catastrophizing appears to be a key intervention target (56). In this study, the scores of rumination and catastrophizing were higher than those of other regulation strategies, suggesting that these strategies should be targeted to improve negative SPA in elderly individuals. Community health care professionals should closely monitor the SPA of elderly individuals with MCC, provide guidance on expressive language exercises, and reduce outward-focused thinking to mitigate the occurrence of alexithymia. Through implementing better coping styles and reducing maladaptive regulation strategies, they can enhance emotion regulation abilities and coping skills, ultimately promoting successful aging and improving the quality of life in their later years.

## 5 Limitations

While we demonstrated a mediating effect of maladaptive cognitive emotion regulation strategies, it is essential to acknowledge certain limitations. First, the data were self-reported, and despite stringent quality control measures, self-report bias may still be present. In the future, this could be assessed in a variety of ways, such as using structured interviews (57). Second, due to cross-sectional and descriptive design, causal inferences could not be made, highlighting the need for future longitudinal studies to validate our findings. Third, our study participants were solely from East China, and representation from Central and West China was lacking. Future research should aim to increase the sample size and diversity to validate our study results. Last, because the model fit was suboptimal when adaptive cognitive emotion regulation strategies and “Self-perceived health status” variables were included, our study only verified the mediating effect of maladaptive cognitive emotion regulation strategies. Future research should expand the sample size to verify the mediating effect of adaptive cognitive emotion regulation strategies and other variables.

## 6 Conclusion

In summary, this study demonstrated that alexithymia may significantly impact the SPA of community-dwelling elderly individuals with MCC through the mediating effect of maladaptive cognitive emotion regulation strategies. Reducing the use of maladaptive cognitive emotion regulation strategies may be an effective strategy for mitigating negative SPA in elderly individuals with MCC, especially those with alexithymia. Nevertheless, future longitudinal research is needed to validate these findings and to consider a broader range of psychosocial factors when exploring the mechanisms of SPA in elderly individuals with MCC.

## Data Availability

The raw data supporting the conclusions of this article will be made available by the authors, without undue reservation.
